# The need for psychological counseling in breast cancer patients before radiotherapy during the COVID-19 pandemic

**DOI:** 10.3389/fpsyg.2022.917175

**Published:** 2022-11-10

**Authors:** Yan-Lin Mo, Xiao-Ying Lai, Min-Feng Mo, Ling Li, Xiao-Dong Zhu

**Affiliations:** ^1^Department of Radiation Oncology, Guangxi Medical University Cancer Hospital, Nanning, China; ^2^Graduate School of Guangxi Medical University, Nanning, China

**Keywords:** COVID-19, psychological counseling, breast cancer, need, depression, anxiety, sleep

## Abstract

The aims of this study were to explore the need for psychological counseling in breast cancer patients before radiotherapy during the COVID-19 pandemic, to distinguish which type of psychological guidance they demanded and to investigate the related factors that could be associated with the need for psychological counseling. A total of 112 eligible patients diagnosed with stage I–IV breast cancer who had received surgery were included. The self-rating depression scale (SDS), self-rating anxiety scale (SAS), Pittsburgh sleep quality index (PSQI), cancer fatigue scale (CFS), and survey for the need for psychological counseling were completed for all subjects prior to radiotherapy. A total of 8.9% and 3.6% of patients suffered from depression and anxiety during the COVID-19 pandemic, respectively. The prevalence of sleep disturbance was 62.5%. Only 12.5% of the patients needed psychological counseling, especially for the type of tumor diagnosis and treatment rather than COVID-19-related protection. The higher the total CFS score was, the lower the need for psychological counseling in breast cancer patients during this pandemic (OR = 0.91, 95% CI = 0.84–0.98). Patients who received 7–8 chemotherapeutic cycles had 6.7 times the risk of needing psychological counseling when compared with those who received 1–6 chemotherapeutic cycles. Fewer breast cancer patients suffered from depression and anxiety before radiotherapy during the COVID-19 pandemic. However, a large number of patients complained of sleep disturbance and fatigue. The majority of patients did not need psychological counseling. More chemotherapeutic cycles or less fatigue could increase their risk of needing psychological counseling, especially for tumor diagnosis and treatment, but not COVID-19-related protection.

## Introduction

Coronavirus disease 2019 (COVID-19) was declared an outbreak by the WHO and rapidly spread worldwide (WHO, 2020). Thus far, more than 618 million people have been diagnosed, and the cumulative number of deaths has exceeded 6 million (WHO, 2022). For fear of contracting viruses, economic pressures and measures of lockdown or social distancing, a growing amount of evidence suggests that the general population experiences higher levels of anxiety, depression, and stress (Salari et al., 2020).

Delays or changes in treatment regimens due to this emergency could increase their concern about the impact on their cancer outcome (Yang et al., 2020). Therefore, cancer patients are at great risk of developing clinical levels of anxiety, depression, sleep disturbance, and fatigue during the COVID-19 pandemic (Miaskowski et al., 2020; Ng et al., 2020; Albano et al., 2021; Gallagher et al., 2021). Even if there is no COVID-19, the prevalence of psychological distress, sleep disturbance, and fatigue is higher in oncology patients than in the general population (Cao et al., 2017). Recent studies showed that 41.7% of breast cancer patients reported insomnia symptoms during the COVID-19 pandemic. A total of 16.7% and 44.4% of patients complained of clinically significant levels of depression and anxiety, respectively (Massicotte et al., 2021). In the high levels of cancer-related and COVID-19 stress group cancer patients, the occurrence rates of sleep disturbance, anxiety, depression, and evening fatigue were 78.0%, 78.0%, 71.2%, and 55.9%, respectively (Miaskowski et al., 2020). These symptom burdens, in turn, may increase a patient’s fear or cause fright and decrease the patient’s quality of life.

Although breast cancer patients could experience higher levels of psychological distress, sleep disturbance, and fatigue during the COVID-19 pandemic (Swainston et al., 2020; Chen et al., 2021; Massicotte et al., 2021), one previous study showed that only 1.6% of cancer patients were seeking psychological help during this pandemic (Wang et al., 2020). It is not clear whether these patients need psychological counseling or what type of psychological guidance these patients need. A limited number of studies have focused on these problems.

Therefore, the aims of this study were to explore the need for psychological counseling in breast cancer patients before radiotherapy during the COVID-19 pandemic and to distinguish which type of psychological guidance they needed. This study also aimed to investigate the related factors that could be associated with the need for psychological counseling in breast cancer patients during this pandemic.

## Materials and methods

### Participants and data collection

Patients who had been diagnosed with stage I to IV breast cancer (8th edition, UICC) and had received surgery and/or chemotherapy, before radiotherapy, were recruited for inclusion in this study at Guangxi Medical University Cancer Hospital. All of the participants were recruited between April and August 2020. The Ethics Committee of Guangxi Medical University Cancer Hospital approved this study. All of the patients provided written informed consent prior to participation.

For patients to be eligible for participation, the following inclusion criteria were used: (a) had received surgery; (b) adult aged 18–65 years; and (c) KPS (Karnofsky performance score) ≥ 80. Patients were excluded if they had two or more cancer diagnoses.

### Instruments and procedures

#### Self-rating depression scale

The Self-rating Depression Scale (SDS) is a questionnaire that is designed to evaluate depression symptoms over the previous week. It contains 20 items and scores from 1 to 4 points for each item. The standardized score is converted from the raw score. In the Chinese version, a standardized score higher than 50 was defined as depression. The test–retest reliability and Cronbach’s alpha coefficient were 0.820 and 0.862, respectively (Dai, 2010).

#### Self-rating anxiety scale

The Self-rating Anxiety Scale (SAS) is a scale designed to evaluate anxiety. It consists of 20 items and scores from 1 to 4 points for each item. In the Chinese version, a standardized score higher than 50 was defined as anxiety. The test–retest reliability and split-half reliability were 0.777 and 0.696, respectively (Dai, 2010).

#### Cancer fatigue scale

The CFS is a scale that is designed to assess self-reported fatigue in cancer patients. It has 15 items and is composed of affective (4 items), cognitive (4 items), and physical (7 items) domains. Each item scores from 1 to 5 points. This instrument has acceptable convergent validity and construct validity. In the current study, the Cronbach’s alpha coefficient of the Chinese version was 0.83 (Shun et al., 2006).

#### Pittsburg sleep quality index

The PSQI (Pittsburg Sleep Quality Index), which has 19 self-rated items, is used to assess the subjective sleep quality. The whole PSQI score has scores from 0 to 21, is the combined of the 7 subscale scores (ranging from 0 to 3): sleep latency, use of sleeping medication, subjective sleep quality, sleep duration, sleep disturbance, daytime dysfunction, and habitual sleep efficiency. A global PSQI score of >5 was considered to be a sleep disturbance. Higher total scores and subscale scores suggested more severe sleep disturbance. The test–retest reliability of the Chinese version is 0.85, and the Cronbach’s alpha coefficient is 0.82–0.83 (Tsai et al., 2005).

Regarding the need for psychological counseling during the COVID-19 pandemic, we designed two items. 1. Do you need psychological counseling during the COVID-19 pandemic? (A: Yes. B: No). If patients choose A, then they will keep going to the next question. 2. Which type of psychological counseling do you prefer? (A: Psychological counseling for tumor diagnosis and treatment. B: Psychological counseling for COVID-19-related protection).

The details of this study were told in full to all patients. They were informed to complete these study questionnaires with the assistance of a trained doctor before radiotherapy in a quiet room. The total assessment duration was approximately 30 min.

#### Statistical analyses

The scores of SDS, SAS, and CFS fit a normal distribution. It is described by the mean. The score of PSQI was not normal. It is described by the median. Chi-square analyses were applied to assess the differences in sleep disturbance, anxiety, and depression between groups with a need for psychological counseling and no need for psychological counseling. The differences in fatigue were evaluated by using *t*-tests. The correlations between variables (age, education, stage, pattern of surgery, recurrence or metastasis, chemotherapy, chemotherapeutic cycle, chemotherapy method, targeted therapy, sleep disturbance, anxiety, depression, and fatigue) and the need for psychological counseling were analyzed by univariate analysis, then the significant factors were included in the multiple-factor analysis. These analyses were two-sided. A *p* < 0.05 indicated statistical significance. SPSS 21.0 (United States) was applied to perform all of the analyses.

## Results

127 patients met all of the criteria in this study. Of these, 15 patients refused to participate because they had a lack of patience (*n* = 3), felt too busy (*n* = 7), or provided no specific reason (*n* = 5). Therefore, 112 patients were included in the final analysis (Table 1).

**Table 1:** The demographic and clinical characteristics of patients (*N* = 112).

Characteristics	*N*	%
Age (mean ± SD)	44.8 ± 8.7	
**Education level**		
Primary/junior school	60	53.6
High school	26	23.2
College or higher	26	23.2
**Stage (8th AJCC)**		
Stage I–II	58	51.8
Stage III–IV	40	35.7
Recurrence or metastasis	14	12.5
**Pattern of surgery**		
Modified radical mastectomy	84	75.0
Breast conserving surgery	28	25.0
**Recurrence or metastasis**		
Yes	14	12.5
No	98	87.5
**Chemotherapy**		
Yes	96	85.7
No	16	14.3
**Chemotherapeutic cycle**		
0–6	22	33.9
7–8	74	66.1
**Chemotherapy method**		
No chemotherapy	16	14.3
Neoadjuvant chemotherapy	38	33.9
Adjuvant chemotherapy	58	51.8
**Targeted therapy**		
Yes	28	25.0
No	84	75.0

SD, Standard Deviation; AJCC, American Joint Committee on Cancer.

The mean age of the patients was 44.8 ± 8.7 years. More than half of the patients (53.6%) had primary or junior school and were stage I–II (51.8%). The percentage of patients with recurrence or metastasis was 12.5%. Seventy-five percent of patients received modified radical mastectomy, while 25% underwent breast-conserving surgery. Most patients had received chemotherapy. Only 14.3% of the patients did not receive chemotherapy. Patients who had 7–8 chemotherapeutic cycles accounted for 66.1%, while 0–6 chemotherapeutic cycles accounted for 33.9%. Patients who received adjuvant chemotherapy, neoadjuvant chemotherapy, and targeted therapy accounted for 51.8%, 33.9%, and 25%, respectively.

The mean SDS and SAS of patients with breast cancer before radiotherapy during the COVID-19 pandemic were 37.7 ± 7.3, 36.8 ± 6.6, respectively (Table 2). The median global PSQI score is 7. A total of 8.9% of patients suffered from depression, and 3.6% suffered from anxiety. The prevalence of sleep disturbance was 62.5%.The mean total CFS score was 20.6 ± 9.2. The mean scores of the physical, affective, and cognitive domains of the CFS were 9.2 ± 4.8, 5.5 ± 3.1, and 5.9 ± 3.3, respectively.

**Table 2:** The scores and the prevalence of depression, anxiety, sleep, and fatigue in patients with breast cancer during COVID-19 pandemic.

Components	Mean ± SD/median	%
SDS	37.7 ± 7.3	8.9
SAS	36.8 ± 6.6	3.6
PSQI		62.5
Subjective sleep quality	1.0	
Sleep latency	2.0	
Sleep duration	0.5	
Habitual sleep efficiency	1.0	
Sleep disturbances	1.0	
Use of sleep medications	0.0	
Daytime dysfunction	1.0	
Global PSQI score	7.0	
CFS		
Total	20.6 ± 9.2	
Physical	9.2 ± 4.8	
Affective	5.5 ± 3.1	
Cognitive	5.9 ± 3.3	

SD, Standard Deviation; SDS, Self-rating Depression Scale; SAS, Self-rating Anxiety Scale; CFS, Cancer Fatigue Scale; PSQI, Pittsburg Sleep Quality Index.

During the COVID-19 pandemic, most breast cancer patients did not need psychological counseling, and only 12.5% (14/112) of patients needed it. Among patients who needed psychological counseling, 85.7% (12/14) preferred psychological counseling for tumor diagnosis and treatment, while 14.3% preferred psychological counseling for COVID-19-related protection. Compared with patients without a need for psychological counseling, patients with a need for psychological counseling reported significantly higher depression, anxiety, and sleep disturbance (all *p* = 0.001, Table 3), as well as more fatigue (*p* = 0.009).

**Table 3:** The prevalence of depression, anxiety, sleep disturbance, and fatigue between patients needed for psychological counseling and no needed for psychological counseling during COVID-19 pandemic.

Components	Psychological counseling		*P*
	Need	No need	
Depression	14.3%	8.2%	0.001
Anxiety	14.3%	2.0%	0.001
Sleep disturbance	42.9%	34.7%	0.001
fatigue(Mean ± SD)	26.6 ± 13.0	19.8 ± 8.2	0.009

SD, Standard deviation.

Variables such as age, education, stage, pattern of surgery, recurrence or metastasis, chemotherapy, chemotherapeutic cycle, chemotherapy method, targeted therapy, sleep disturbance, anxiety, depression, and fatigue were all considered to be potential factors associated with the need for psychological counseling. Univariate analysis showed that the chemotherapeutic cycle, anxiety, and fatigue were significantly associated with the need for psychological counseling (Table 4). Therefore, these factors were included in the multivariate analysis. Binary logistic regression analyses indicated that the higher the total CFS score was, the less need for psychological counseling in breast cancer patients during this pandemic(OR = 0.91, 95% CI = 0.84–0.98). Patients who received 7–8 chemotherapeutic cycles had 6.7 times the risk of needing psychological counseling (95% CI = 1.66–27.01) when compared with those who received 1–6 chemotherapeutic cycles.

**Table 4:** The predictors of need for psychological counseling in breast cancer patients during COVID-19 pandemic.

Variable	Univariate analysis		Multivariate analysis	
	OR(95% CI)	*P*	OR(95% CI)	*P*
Age	0.96(0.90–1.03)	0.248		
Education	1.68(0.76–3.70)	0.202		
Stage	1.09(0.48–2.46)	0.838		
Pattern of surgery	0.81(0.23–2.82)	0.742		
Recurrence or metastasis	0.00	0.999		
Chemotherapy	1.00(0.20–4.96)	1.000		
Chemotherapeutic cycle	3.02(0.97–9.38)	0.056	6.70(1.66–27.01)	0.008
Chemotherapy method	1.21(0.57–2.57)	0.621		
Targeted therapy	2.59(0.81–8.27)	0.108		
Sleep disturbance	0.40(0.13–1.24)	0.113		
Anxiety	8.00(1.03–62.12)	0.047	3.91(0.35–44.24)	0.271
Depression	1.88(0.36–9.89)	0.459		
Total CFS score	0.92(0.86–0.98)	0.012	0.91(0.84–0.98)	0.016

OR, Odds Ratio; 95% CI, 95% Confidence; CFS, Cancer Fatigue Scale.

## Discussion

The current COVID-19 pandemic is considered to be an extremely stressful event that could result in increased psychological symptoms, sleep disturbance, and fatigue in cancer patients. Findings from this study suggested that fewer breast cancer patients suffered from depression and anxiety before radiotherapy during the COVID-19 pandemic. However, nearly two-thirds of patients reported sleep disturbance. Only 12.5% of patients (higher depression, anxiety, sleep disturbance, and fatigue) expressed their need for psychological counseling, especially for the type of tumor diagnosis and treatment. Patients who receive more chemotherapeutic cycles (7–8 cycles) could have an increased risk of needing psychological counseling, while patients with higher total CFS scores have less need for psychological counseling.

Studies have shown that patients with breast cancer are at great risk of suffering from depression and anxiety during the COVID-19 pandemic (Chen et al., 2021; Kim and Kim, 2021; Massicotte et al., 2021). However, this finding contradicts the finding from our study that fewer breast cancer patients suffered from these psychological symptoms during that time. Only 8.9% and 3.6% of patients suffered from depression and anxiety, respectively. One reason for the inconsistent results could be associated with the different survey times between these studies. This study was conducted from April 2020 to August 2020 in China. The COVID-19 pandemic has been almost completely controlled during this time. However, other studies have performed investigations during disease outbreaks and epidemics. The depression and anxiety of patients could be alleviated with the outbreak under control. Unlike depression and anxiety, 62.5% of patients complained of sleep disturbance in this study. Our result is higher than those of previous studies in which the prevalence of insomnia ranged from 21.6% to 41.7% (Chen et al., 2021; Massicotte et al., 2021). There was evidence that 78% of cancer patients reported sleep disturbance in the stressed group, and the occurrence rate for evening fatigue was 55.9% (Miaskowski et al., 2020). The high prevalence of sleep disturbance could be associated with high levels of cancer-related stress and COVID-19. However, another study showed that young adult cancer survivors had less insomnia severity during the pandemic (Zhou et al., 2020).

For different degrees of fear about infecting COVID-19 and the impact on cancer care by this epidemic, providing psychological support during the COVID-19 pandemic could relieve patients’ depression, anxiety, sleep disturbances, and fatigue. However, in this study, only 12.5% of the patients needed psychological counseling. There was a study similar to ours in which only 1.6% of cancer patients sought psychological counseling during this time (Wang et al., 2020). Findings explored in patients with colorectal cancer showed that the majority thought they did not need support (including mental health) during the COVID-19 pandemic (Kamposioras et al., 2021). However, our result is lower than that of a previous study that investigated cancer patients and their relatives in Wuhan, in which 40.2% of the subjects needed psychosocial support during this period (Yang et al., 2021). One reason for the higher need for psychological counseling in that study could be related to the subjects who were chosen (which included the patients’ relatives), while all of the subjects in the current study were patients with cancer. Another reason could be the different types of cancer patients between the two studies. Their study included patients with other cancers. Our study also suggested that breast cancer patients who receive more chemotherapeutic cycles could have an increased risk of needing psychological counseling. There is evidence that breast cancer patients with more cycles of chemotherapy have worse sleep and more depressive symptoms compared to baseline levels (Ancoli-Israel et al., 2014). These increased depressive symptoms and worse sleep could increase their need for psychological counseling. In turn, the findings from our study also showed that patients who needed psychological counseling reported significantly higher depression, anxiety, sleep disturbances and fatigue than those who did not need psychological counseling. An interesting finding also found in this study was that the higher the total CFS score was, the lower the need for psychological counseling in breast cancer patients during this pandemic. The exact reason for this correlation is unclear. Additional study is needed to elucidate the relationship between fatigue and the need for psychological counseling.

Regarding the type of psychological counseling during the COVID-19 pandemic, the majority of breast cancer patients (who needed psychological counseling) preferred psychological counseling for tumor diagnosis and treatment, while 14.3% of patients preferred psychological counseling for COVID-19-related protection. A previous study investigated patients with gynecological cancer and showed that only 17.5% of the patients were more worried about COVID-19 than about their cancer disease (Gultekin et al., 2021). This result is similar to our finding. Compared to COVID-19, most patients were likely to adhere to the schedules of oncology treatment and surveillance. They were more concerned about whether their treatment plan was delayed or changed and whether the cancer disease would recur or metastasize. These factors could be associated with an increase in psychological distress (Chen et al., 2021; Kim and Kim, 2021) and, in turn, could increase their need for psychological counseling.

### Limitations

This study is a descriptive study. By design, the study had inadequate power to determine the causal relationships among chemotherapeutic cycle, fatigue, or other collected values and the need for psychological counseling. Second, this study was conducted in China at the time that COVID-19 was almost completely controlled. Compared with the time of COVID-19 outbreaks and epidemics, the prevalence of depression, anxiety, sleep disturbances and fatigue and the need for psychological counseling could have some differences. However, at present, with the wide use of vaccines, the COVID-19 pandemic has been largely controlled worldwide. This situation is similar to our study. Our results could provide some information about the prevalence of psychological distress, sleep disturbances and fatigue and the need for psychological counseling at this time. Third, the cross-sectional design and the temporal relation between the scores and the outcomes cannot be established. It is also limited by a single center experience.

This study suggests that fewer breast cancer patients suffered from depression and anxiety before radiotherapy during the COVID-19 pandemic. However, a large number of patients complained of sleep disturbance and fatigue. The majority of patients did not need psychological counseling. More chemotherapeutic cycles or less fatigue could increase their risk of needing psychological counseling, especially for tumor diagnosis and treatment, but not COVID-19-related protection. More randomized, controlled studies are recommended to further explore their need for psychological counseling and to determine which type of psychological guidance they need during or after the COVID-19 pandemic.

## Data availability statement

The original contributions presented in the study are included in the article/Supplementary material, further inquiries can be directed to the corresponding authors.

## Ethics statement

The studies involving human participants were reviewed and approved by Guangxi Medical University Cancer Hospital. The patients/participants provided their written informed consent to participate in this study.

## Author contributions

Y-LM and X-DZ designed the conception and the work. Y-LM, M-FM, X-YL, and LL acquired and analyzed the data and prepared table. Y-LM, LL, and X-DZ drafted this work. All authors contributed to the article and approved the submitted version.

## Conflict of interest

The authors declare that the research was conducted in the absence of any commercial or financial relationships that could be construed as a potential conflict of interest.

## Publisher’s note

All claims expressed in this article are solely those of the authors and do not necessarily represent those of their affiliated organizations, or those of the publisher, the editors and the reviewers. Any product that may be evaluated in this article, or claim that may be made by its manufacturer, is not guaranteed or endorsed by the publisher.
